# Economic costs associated with insomnia in adults with subthreshold depression or major depressive disorder

**DOI:** 10.1192/bjo.2025.10077

**Published:** 2025-07-21

**Authors:** Phuong Hong Le, Long Khanh-Dao Le, Joahna Kevin Perez, Shantha M. W. Rajaratnam, Cathrine Mihalopoulos

**Affiliations:** Health Economics Group, School of Public Health and Preventive Medicine, Monash University, Melbourne, Australia; School of Psychological Sciences and Turner Institute for Brain and Mental Health, Monash University, Clayton, Australia

**Keywords:** Cost, comorbidity, depression, insomnia, subsyndromal depression

## Abstract

**Background:**

Previous studies have found substantial costs to be associated with depression and insomnia (as separate entities).

**Aims:**

To estimate healthcare service use and costs associated with insomnia in Australian adults experiencing subthreshold depression or major depressive disorder (MDD).

**Method:**

Healthcare service use and productivity loss were extracted from the cross-sectional 2020–2022 National Survey of Mental Health and Wellbeing data. Insomnia and depression were assessed using questions aligned with DSM-IV criteria. Weighted two-part models were used to calculate average annual costs (presented as 2021–2022 Australian dollars).

**Results:**

The analytical sample meeting subthreshold depression or MDD criteria consisted of 1331 adults (aged 40.5 ± 16.1 years; 59% female; insomnia prevalence: 84%). Healthcare service use and healthcare costs between individuals with insomnia and those without insomnia were similar in the MDD group. For subthreshold depression, healthcare costs were significantly higher for those with insomnia compared with those without insomnia (*Δ* = A$990, 95% CI: 234 to 1747), but healthcare resource use was not significantly different. Productivity loss among employed people and reduced employment were much greater (although the difference did not reach statistical significance) in adults with insomnia compared with those without insomnia.

**Conclusions:**

Healthcare resource use among adults with depression was similar in those with and without insomnia. However, higher healthcare costs associated with insomnia were observed in adults with subthreshold depression. Further studies are encouraged to understand the nature of the increased healthcare cost associated with insomnia in individuals with subthreshold depression and to optimise healthcare service use in people with comorbid depression and insomnia.

Depression is among the top 25 leading causes of burden of disease as measured by disability-adjusted life years (DALYs).^1^ Furthermore, total healthcare and societal costs for depression have been estimated to be Australian dollars (A$) 129.7 million and A$1496 million per year in Australia (expressed in 2021–2022 A$).^2^ A study in the US context also showed high societal costs associated with major depressive disorder (MDD), amounting to A$508 billion or A$25 655 per adult with MDD (expressed in 2021–2022 A$), with productivity costs accounting for 61.9% of these total costs.^3^ Subthreshold depression, also known as subsyndromal depression, has been increasingly studied over the past decade.^4^ Despite having symptoms of depression that do not meet the full diagnostic criteria for MDD, people with subthreshold depression have reduced quality of life and increased healthcare resource use compared with those without depression.^5,6^

Insomnia is a common symptom of depressive disorders^7^ and has been found to have the highest economic costs among high-prevalence sleep disorders. For example, in the US, it has been estimated that insomnia can incur up to A$20.3 billion in healthcare and societal costs (expressed as 2021–2022 A$).^8^ Recently, evidence-based recommendations have stated that insomnia treatment should be added to standard depression care for those with comorbid insomnia and depression.^9,10^ Understanding the current healthcare and societal costs of individuals with subthreshold depression or MDD who experience insomnia symptoms compared with those without is essential for consideration of the balance between cost and clinical effectiveness when implementing a new intervention in current practice. However, the costs associated with comorbid insomnia and depression have been understudied. In this world-first study, we aimed to estimate the economic costs and healthcare resource use associated with insomnia in Australian adults with subthreshold depression and MDD using a nationally representative survey.

## Method

### Study design

Data were derived from the cross-sectional Australian National Survey of Mental Health and Wellbeing (NSMHWB) 2020–2022 (*N* = 15 893; response rate: 52%). The NSMHWB is a population-based representative survey that contains comprehensive data on diagnoses of high-prevalence mental disorders, including depression, anxiety and substance use disorders, as well as sociodemographic characteristics, comorbid physical conditions, and healthcare resource use and productivity loss due to mental health issues. The study design and the methodology of the survey have been described in detail elsewhere.^11^

### Population

Economic costs associated with insomnia were calculated for Australians aged 18+ years who experienced subthreshold depression or MDD within the past 12 months. Although the NSMHWB survey offered diagnoses for mental disorders over 30-day, 12-month and lifetime periods, 12-month diagnosis was selected to align with the data on healthcare resource use collected over the past 12 months. The process by which the analytic sample was selected is illustrated in Supplementary Fig. 1 available at https://doi.org/10.1192/bjo.2025.10077. MDD was defined using the World Health Organization World Mental Health-Composite International Diagnostic Interview (WMH-CIDI) 3.0 based on DSM-IV criteria with hierarchy rules applied.^11^ Subthreshold depression was defined as experiencing a core symptom of depression over the past 12 months, including feeling sad, empty, depressed or discouraged about life, or loss of interest in most activities, while not meeting the diagnostic criteria for MDD.

Insomnia was characterised as complaints of difficulty initiating or maintaining sleep for more than half the time of the most severe depressive episode in the last 12 months. Difficulty initiating sleep was derived from a self-reported sleep latency of more than 30 min using a four-point scale (‘Never took longer than 30 minutes to fall asleep’; ‘Took at least 30 minutes to fall asleep, less than half the time’; ‘Took at least 30 minutes to fall asleep, more than half the time’; and ‘Took more than 60 minutes to fall asleep, more than half the time’). Difficulty maintaining sleep was defined as being awake more than once a night for more than 20 min after sleep onset or waking up more than 30 min before the scheduled time using two question scored on a four-point scale. These questions were only asked to survey participants who reported that they experienced sadness, emptiness, depression or discouragement about life or a loss of interest in most activities (subthreshold depression).

### Cost analyses

In this study, we used a bottom-up approach to calculate economic costs. For the analysis from a healthcare perspective, we considered costs of consultations (including consultations delivered by general practitioners, psychiatrists, psychologists, mental health nurses and other health practitioners), hospital admissions and medications for mental disorders (as asked by the survey). Costs of productivity loss due to absenteeism and presenteeism for employed people were included when the societal perspective was adopted.

Respondents self-reported quantity, average length and out-of-pocket costs of mental-health-related consultations with health practitioners. Each consultation reimbursed by Medicare or private health insurance was assigned the unit cost adjusted by the type of healthcare professional and length of the consultation. In the base case analysis, the unit cost for consultations was the weighted average cost of the Medicare Benefits Schedule (MBS) standard professional attendance items.^12^ The unit cost that was estimated using all MBS items for mental health was applied in sensitivity analysis 1. Any out-of-pocket costs reported by the participants were also included.

The survey participants were asked about their number of hospital admissions, reasons for hospital admissions and number of nights they stayed in the hospital for mental health issues. Costs of hospital admissions were determined using the 2021–2022 National Efficient Price (A$5597) multiplied by the National Weighted Activity Unit (NWAU).^13^ The National Efficient Price is determined by the Independent Hospital Pricing Authority annually. The NWAU was defined using Australian refined diagnosis-related groups for hospital admission reasons reported by the participants based on Independent Hospital Pricing Authority algorithms.^13^ If multiple reasons for hospital admissions were reported, hospital admission cost was the average of costs across different NWAUs applied in the base case analysis. The maximum and minimum values were used instead in the sensitivity analyses (SA2 and SA3). The NSMHWB data owner created data items for mental-health-related medications (i.e. antipsychotics, anxiolytics, hypnotics and sedatives, antidepressants, psychostimulants and agents used for attention-deficit hyperactivity disorder, and nootropics) used in the past 12 months (dichotomous values) for each survey participant by linking NSMHWB records to Pharmaceutical Benefits Scheme (PBS) data.^11^ The PBS is Australia’s publicly subsidised national formulary and contains information on all medicines that are prescribed by doctors and ultimately purchased by patients that are listed on the PBS (7.2% of the analytic sample of this study could not be linked to PBS data). Weighted unit costs per person for each medication type were derived from the 2021–2022 reports by the Australian Institute of Health and Welfare (2023)^14^ and PBS.^15^

With respect to productivity loss for employed people, the number of days in the past year that respondents were totally unable to work and the amount of interference with their ability to work on a ten-point scale (with higher scores indicating more interference) were collected in the NSMHWB. We used the human capital approach^16^ to estimate the cost related to productivity losses using the Australian Bureau of Statistics (ABS)^17^ weekly earnings in Australia adjusted by age and sex in the base case analysis. Sensitivity analysis was conducted by applying earnings adjusted by industry, age and sex (SA4).^17^ All costs are presented as 2021–2022 Australian dollars.

### Statistical analysis

All analyses were performed using R software version 4.3.0 on the DataLab platform.^18^ Owing to the skewed nature and substantial proportion of zero values of cost data, two-part hurdle models adjusted by covariates (i.e. age, sex, comorbid mental health conditions, comorbid physical conditions, education level, marital status and household socioeconomic indices) and the interaction term between insomnia and depression were used to estimate healthcare and societal costs (including and excluding the productivity loss of being not employed) associated with insomnia in the subgroups with subthreshold depression and MDD. Part 1 was a logistic regression model to generate probabilities of generating the costs, and part 2 was a gamma-log generalised linear model to calculate costs (if any) incurred. For costs associated with productivity loss for employees (including presenteeism, absenteeism and total productivity loss), the two-part models were applied for employed people. A logistic regression was used to estimate the probability of being employed for each subgroup. The tests used to select the gamma distribution, the log link function and the covariates included in the final models are described in the supplementary material. Survey weights sourced from the NSMHWB data were applied to all models. The weighting approach and calibration variables used have been described in detail elsewhere.^11^

Healthcare resource use refers to the frequency of use of healthcare services, including consultations, hospital admissions and medication use. The expected probabilities of using these services were calculated from the first part of the statistical models. Annual healthcare and societal costs per person and excess costs associated with insomnia in adults with MDD and those with subthreshold depression (i.e. the mean differences in costs between individuals with versus without insomnia) were the product of the expected probability of incurring costs and the expected cost (whereby unit costs were added to the service use) estimated from part 1 and part 2 of the models, with the standard errors of the annual costs estimated using 1000 bootstrapped iterations.

A full description of all analytic variables is presented in Supplementary Table 1. Percentages of missing values for all variables were less than 11%. Pairwise deletion was applied to deal with missing values. All analyses were implemented using R version 4.3.0 on the DataLab platform.^18^ The R code was adjusted from that provided by Zhou et al (2024)^19^ to be appropriate for the data-set and context of this study.

### Sensitivity and subgroup analyses

The sensitivity of our findings was tested by changing unit costs of consultations, hospital admission and productivity loss as mentioned above (sensitivity analyses 1–4). In sensitivity analysis 5, productivity loss for people who were not employed was also incorporated into the societal cost. The NSMHWB survey collected information on reasons for not working among individuals who were not employed or not studying using a multiple-response question.

Productivity losses for unemployment and labour force absence were estimated for those who did not work owing to their own ill health or disability and were aged between 18 and 64 years, as follows: productivity losses by not being employed = Australian participation rate × (1 − Australian unemployment rate) × age- and sex-adjusted weekly earnings × length of not being employed (weeks). The underlying theory of this approach was that people who did not work owing to a health condition would have the same employment ratio as the general population.^20^ This formula has been applied in previous costing studies conducted in Australian contexts.^21,22^ Participation rate, unemployment rate, and age- and sex-adjusted weekly earnings of the Australian general population were derived from an ABS report.^17,23^ In terms of the length of not being employed, we used 48 weeks on the basis of the recommended annual leave in Australia (four weeks per year for full-time and part-time employees) to calculate productivity losses for individuals who were not in the labour force,^24^ assuming they were out of the labour force during the entire year (unfortunately, the NSMHWB data did not include the number of weeks since last work for those who were not in the labour force). For unemployed people, we used the reported number of weeks since they last worked instead if it was less than 48 weeks.

We repeated the base case analysis, categorising people experiencing MDD into two severity levels of MDD (mild to moderate and severe) using an Australian version of the WMH-CIDI.^11^ The Australian version was made by adjusting the WMH-CIDI version 3.0 and the New Zealand version to include both DSM-IV and ICD-10 calculations. The details of the differences across all versions are presented by the ABS (2024).^11^

### Consent statement

This study used secondary de-identified NSMHWB data. The NSMHWB is part of the wider Intergenerational Health and Mental Health Study funded by the Australian Government Department of Health and Aged Care. Owing to the sensitivity of the mental health topic, data collection was conducted on a voluntary basis.^11^

### Ethics statement

This study was granted low-risk ethics approval from the Monash University Human Research Ethics Committee (project ID: 36681).

## Results

### Demographic characteristics

The analytic sample size was 1331 adults (aged 40.5 ± 16.1 years, female: 59%), with 32.4% having subthreshold depression and 67.6% having MDD. The weighted percentage of individuals who experienced insomnia symptoms was slightly lower in the subthreshold depression population compared with the MDD population (81.3% *v*. 87.6%). Descriptive characteristics of all analytic variables are presented in Table 1.

**Table 1:** Descriptive statistics^a^

	Subthreshold depression		Major depressive disorder	
Characteristics	Insomnia: no	Insomnia: yes	Insomnia: no	Insomnia: yes
Sample, *N*	77	337	135	782
Population (×1000), *N*	102.4	445.58	141.64	1,001.25
Age in years, weighted mean (s.d.)	39.7 (17.0)	40.7 (16.1)	39.3 (15.5)	40.6 (16.0)
Age group, *n* (weighted %)				
Adults	63 (88.4)	294 (89.3)	124 (91.7)	683 (91.9)
Older adults	14 (11.6)	43 (10.7)	11 (8.3)	99 (8.1)
Sex, *n* (weighted %)				
Male	27 (39.8)	129 (37.3)	53 (41.9)	284 (41.9)
Female	50 (60.2)	208 (62.7)	82 (58.1)	498 (58.1)
Indigenous, *n* (weighted %)				
Yes	NR	NR	NR	NR
No	NR	NR	NR	NR
Marital status, *n* (weighted %)				
Non-partnered	47 (51.1)	214 (58.2)	83 (58.1)	511 (58.1)
Partnered	30 (48.9)	123 (41.8)	52 (41.9)	271 (41.9)
Residency status, *n* (weighted %)				
Australian or permanent resident	NR	NR	NR	NR
Temporary resident	NR	NR	NR	NR
English as the main language, *n* (weighted %)				
Yes	NR	NR	NR	NR
No	NR	NR	NR	NR
Education level, *n* (weighted %)				
High	28 (30.7)	102 (21.6)	56 (37.3)	284 (31.0)
Intermediate or low	49 (69.3)	235 (78.4)	79 (62.7)	498 (69.0)
Employment status, *n* (weighted %)				
Employed	56 (75.5)	212 (57.8)	105 (73.7)	519 (63.3)
Unemployed or not in the labour force	21 (24.5)	125 (42.2)	30 (26.3)	263 (36.7)
SEIFA quintile (disadvantage, national level), *n* (weighted %)				
First	14 (18.2)	75 (22.3)	15 (11.4)	113 (14.5)
Second or third	29 (37.7)	147 (43.7)	59 (43.5)	334 (42.8)
Fourth or fifth	34.1 (44)	11438 (34)	61 (45.0)	334 (42.7)
SEIFA score, weighted mean (s.d.)	5.61 (2.75)	5.67 (3.11)	5.11 (2.78)	5.85 (2.59)
Depression severity				
Mild to moderate	Not applicable	Not applicable	98 (72.6)	425 (54.4)
Severe	Not applicable	Not applicable	37 (27.4)	357 (45.6)
Comorbid physical condition, *n* (weighted %)				
No	52 (75.4)	201 (58.5)	89 (67.3)	464 (61.6)
Yes	25 (24.6)	136 (41.5)	46 (32.7)	318 (38.4)
Comorbid mental condition, *n* (weighted %)				
No	35 (44.6)	102 (27.3)	52 (38.0)	247 (30.2)
Yes	42 (55.4)	235 (72.7)	83 (62.0)	535 (69.8)

SEIFA, Socio-Economic Indexes for Areas.

a. NR indicates that the number of people who were Indigenous Australians, temporary residents, or did not speak English as the main language was below ten. The exact numbers could not be reported on the basis of the rule of ten of the data owner (Australian Bureau of Statistics).^18^

### Healthcare resource use and economic costs associated with insomnia

Multicollinearities between insomnia and depression and between insomnia and comorbid mental conditions were not issues in this study (insomnia and depression: *P* = 0.088; insomnia and comorbid mental health conditions: *P* = 0.02; Cramer’s *V* = 0.06; small effect).

### Healthcare resource use

Table 2 shows healthcare resource use for subgroups of participants categorised by depression and insomnia. In the subthreshold depression population, people with insomnia symptoms did not show significant differences in their probabilities of consultations (odds ratio 1.35, *P* = 0.25), hospital admissions (odds ratio 2.79, *P* = 0.32) or medication use (odds ratio 1.46, *P* = 0.16) compared with those without. In the MDD population, people with and without insomnia had similar patterns of healthcare resource use (consultations: odds ratio 1.09, *P* = 0.69; hospital admission: odds ratio 1.10, *P* = 0.91; medication: odds ratio 0.97, *P* = 0.88).

**Table 2:** Health service use, absenteeism and presenteeism associated with insomnia derived from statistical models

Depression type	Insomnia	Probability of using services (95% CI)	Odds ratio (s.e.)	*P*-value
Consultations				
Subthreshold depression	No	0.498 (0.385–0.610)		
	Yes	0.572 (0.512–0.630)	1.348 (0.347)	0.246
MDD	No	0.580 (0.483–0.672)		
	Yes	0.601 (0.561–0.640)	1.090 (0.231)	0.685
Hospital admissions				
Subthreshold depression	No	0.009 (0.001–0.066)		
	Yes	0.024 (0.009–0.059)	2.788 (2.893)	0.323
MDD	No	0.010 (0.002–0.053)		
	Yes	0.011 (0.005–0.028)	1.104 (0.926)	0.906
Medications				
Subthreshold depression	No	0.428 (0.318–0.539)		
	Yes	0.517 (0.449–0.585)	1.457 (0.393)	0.163
MDD	No	0.520 (0.404–0.635)		
	Yes	0.511 (0.468–0.555)	0.966 (0.214)	0.875

Depression	Insomnia	Probability of productivity loss (95% CI)	Odds ratio (s.e.)	*P*-value
Absenteeism				
Subthreshold depression	No	0.491 (0.348–0.634)		
	Yes	0.640 (0.567–0.713)	1.949 (0.598)	0.030*
MDD	No	0.589 (0.480–0.697)		
	Yes	0.603 (0.554–0.652)	1.067 (0.267)	0.795
Presenteeism				
Subthreshold depression	No	0.760 (0.652–0.868)		
	Yes	0.863 (0.824–0.903)	2.031 (0.781)	0.065
MDD	No	0.883 (0.838–0.928)		
	Yes	0.846 (0.814–0.878)	0.724 (0.276)	0.396

MDD, major depressive disorder.

*P < 0.05

### Healthcare costs

Table 3 presents probabilities of incurring costs and average annual costs for each subgroup of individuals with insomnia and depression. The average annual healthcare cost per person with comorbid insomnia and depression symptoms was A$1961 (95% CI: 1471 to 2451) for the subthreshold depression population and A$1686 (95% CI: 1235 to 2136) for the MDD populations. Insomnia was found to be significantly associated with higher average annual healthcare costs in people with subthreshold depression (mean difference A$990, 95% CI: 234 to 1747), whereas healthcare costs for MDD adults with and without insomnia were nearly equal (mean difference A$61, 95% CI: −929 to 1050). The significant difference in healthcare cost between insomnia and non-insomnia groups observed in the subthreshold population was primarily due to the significantly higher cost of consultations and hospital admissions per person seeking these healthcare services (A$2,896 (95% CI: 2207 to 3585) versus A$1,297 (95% CI: 647 to 1947)) (Supplementary Table 5).

**Table 3:** Average annual healthcare and societal costs associated with insomnia in people with subthreshold depression and those with MDD derived from statistical models

Depression type	Insomnia	Probability of incurring costs (95% CI)	Costs if any incurred A$ (95% CI)	Average cost per person A$ (95% CI)	Mean difference A$ (95% CI)
Healthcare cost					
Subthreshold depression	No	0.637 (0.490, 0.785)	1496 (410, 2582)	971 (387, 1555)	
	Yes	0.681 (0.612, 0.749)	2834 (2098, 3570)	1961 (1471, 2451)	990 (234, 1747)^a^
MDD	No	0.745 (0.634, 0.855)	2151 (792, 3511)	1625 (641, 2609)	
	Yes	0.727 (0.692, 0.762)	2285 (1677, 2892)	1686 (1235, 2136)	61 (−929, 1050)
Probability of being employed					
Subthreshold depression	No	0.765 (0.697, 0.834)			
	Yes	0.663 (0.607, 0.720)			
MDD	No	0.776 (0.698, 0.855)			
	Yes	0.704 (0.679, 0.730)			
Productivity loss cost for employed people					
Subthreshold depression	No	0.766 (0.662, 0.871)	18 059 (9687, 26 430)	13 153 (5225, 21 081)	
	Yes	0.874 (0.834, 0.913)	21 007 (17 629, 24 384)	17 845 (14 744, 20 945)	4692 (−3813, 13 196)
MDD	No	0.907 (0.867, 0.948)	18 244 (11 076, 25 411)	16 215 (9357, 23 073)	
	Yes	0.896 (0.874, 0.918)	25 182 (20 958, 29 407)	22 045 (18 049, 26 042)	5830 (−1541, 13 202)
Societal cost					
Subthreshold depression	No	0.809 (0.707, 0.911)	12 806 (3414, 22 198)	10 463 (2504, 18 423)	
	Yes	0.869 (0.833, 0.905)	12 079 (9993, 14 166)	10 570 (8643, 12 496)	107 (−8026, 8239)
MDD	No	0.887 (0.788, 0.986)	13 257 (2417, 24 097)	11 829 (2344, 21 313)	
	Yes	0.884 (0.859, 0.909)	13 458 (11 107, 15 810)	11 967 (9834, 14 099)	138 (−9711, 9987)

A$, Australian dollars. MDD, major depressive disorder.

a. 95% CI not including the null value (zero), indicating a statistically significant difference.

### Productivity loss for employed people

The probability of employment was lower for those with insomnia than those without insomnia in both the subthreshold depression [0.66 (95% CI: 0.61 to 0.72) versus 0.77 (95% CI: 0.70 to 0.83), *P* = 0.07] and MDD [0.70 (95% CI: 0.680 to 0.73) versus 0.78 (95% CI: 0.70 to 0.86), *P* = 0.09] populations. For the subthreshold population, employed adults experiencing insomnia symptoms had two-fold more absences from work (odds ratio 2.18, *P* = 0.03) and higher presenteeism probability (odds ratio 2.03, *P* = 0.07). For the MDD population, adults with or without insomnia had a similar probability of absenteeism (odds ratio 1.07, *P* = 0.26) and a lower probability of presenteeism (odds ratio 0.72, *P* = 0.40) (Table 2).

The average annual cost of productivity loss was substantial in employed people with comorbid depression and insomnia symptoms, amounting to A$17 845 (95% CI: 14 744 to 20 945) for the subthreshold depression population and A$22 045 (95% CI: 18 049 to 26 042) for the MDD population. The difference in cost of productivity loss in employed adults with insomnia, compared with those without, was higher but did not reach statistical significance for either subthreshold depression (mean difference: A$4692, 95% CI: −3813 to 13 196) or MDD (mean difference: A$5830, 95% CI: −1541 to 13 202) (Table 3).

### Societal cost

The difference in average annual societal cost between those with and without insomnia was not statistically significantly different for either the subthreshold depression (mean difference: A$107, 95% CI: −8026 to 8239) or the MDD (mean difference: A$138, 95% CI: −9711 to 9987) population (Table 3).

Supplementary Tables 2–4 present the outcomes of regression models for healthcare, societal and productivity loss costs adjusted by selected covariates. The average annual costs of all cost components are fully presented in Supplementary Table 5.

### Sensitivity analyses by changing unit costs

The results of the sensitivity analyses are shown in Table 4. Changing values of unit costs for consultations and hospital admission (sensitivity analyses 1–3) did not considerably affect the results. In sensitivity analysis 4, when the work industry was factored in to determine the unit cost for weekly earnings, the mean difference in productivity loss costs for the subthreshold depression population substantially decreased (A$2781, 95% CI: −5620 to 11 181). The mean difference in societal cost was also affected as a result. Compared with the results of the base case analysis, a larger value was observed in the MDD population A$1077 (95% CI: −5791 to 7945), whereas the value for the subthreshold depression population became negative, indicating that individuals without insomnia generated higher productivity costs (mean difference: −A$841, 95% CI: −9358 to 7675).

**Table 4:** Sensitivity analyses changing unit costs: average cost per person (A$, 95% CI)

Depression	Insomnia	Sensitivity analysis 1: MBS unit cost changed			Sensitivity analysis 2: maximum value of hospital admission unit cost			Sensitivity analysis 3: minimum value of hospital admission cost			Sensitivity analysis 4: ABS weekly earnings adjusted by age, sex, and industry		Sensitivity analysis 5: incorporating productivity loss of not-employed people in societal cost	
		Average cost per person	Mean difference		Average cost per person	Mean difference		Average cost per person	Mean difference		Average cost per person	Mean difference	Average cost per person	Mean difference
Healthcare cost														
Subthreshold depression	No	1028 (421, 1636)			988 (396, 1580)			948 (391, 1504)						
	Yes	2006 (1514, 2498)	978 (202, 1754)^a^		2069 (1527, 2612)	1082 (255, 1909)^a^		1833 (1357, 2310)	886 (133, 1638)^a^					
MDD	No	1660 (639, 2682)			1643 (624, 2662)			1604 (626, 2582)						
	Yes	1758 (1303, 2212)	97 (−934, 1128)		1730 (1272, 2189)	87 (−954, 1128)		1646 (1211, 2081)	42 (−955, 1040)					
Productivity loss cost														
Subthreshold depression	No										11 913 (3957, 19 869)			
	Yes										14 694 (12 128, 17 260)	2781 (−5620, 11 181)		
MDD	No										13 953 (8365, 19 541)			
	Yes										19 474 (15723, 23226)	5521 (−820, 11 861)		
Societal cost														
Subthreshold depression	No	10 536 (2610, 18 463)			10 480 (2504, 18 455)			10 443 (2502, 18 385)			10 159 (1735, 18 583)		12 816 (6826, 18 805)	
	Yes	10 621 (8695, 12 548)	85 (−8018, 8188)		10 682 (8744, 12 620)	202 (−7949, 8354)		10 430 (8515, 12 345)	−13 (−8128, 8101)		9318 (7632, 11 003)	−841 (−9358, 7675)	18 293 (15 686, 20 900)	5478 (−1020, 11 975)
MDD	No	11861 (2381, 21 342)			11 834 (2365, 21 303)			11 818 (2319, 21 316)			9835 (3308, 16 362)		14 194 (8680, 19 707)	
	Yes	12031 (9898, 14 163)	169 (−9678, 10 016)		12 019 (9882, 14 155)	185 (−9648, 10 018)		11 922 (9793, 14 051)	104 (−9756, 9965)		10 912 (8762, 13 062)	1077 (−5791, 7945)	18 075 (15 808, 20 343)	3882 (−2012, 9775)

A$, Australian dollars. ABS, Australian Bureau of Statistics; MBS, Medicare Benefits Schedule; MDD, major depressive disorder.

a. 95% CI not including the null value (zero), indicating a statistically significant difference.

When the productivity loss of not-employed people was included in the societal cost, the difference in societal costs between those with and without insomnia became much larger, although it remained not significant (subthreshold depression: A$5478, 95% CI: −1020 to 11 975; MDD: A$3882, 95% CI: −2012 to 9775) (refer to Table 4, sensitivity analysis 5). The proportion of individuals not working owing to their own illnesses or disabilities was two times as high among those experiencing insomnia symptoms compared with those without insomnia symptoms in both the subthreshold depression and MDD populations.

### Subgroup analyses for depression severity levels

When study participants were categorised by depression severity level, 43.4% of those with MDD were classified into the severe group. We also used the World Mental Health 3.0 and New Zealand versions but found no substantial differences compared with the application of the Australian version. The presence of insomnia showed a significant association with increasing levels of depression severity (*χ* ^2^ = 11.73, *P* < 0.01, Cramer’s *V* = 0.07). The proportions of individuals experiencing insomnia symptoms in the mild to moderate and severe depression subgroups were 84.1 and 92.1%, respectively.

The effects of depression severity on economic costs are shown in Table 5 (regression models in Supplementary Tables 6 and 7). For mild to moderate MDD, adults with insomnia symptoms had higher healthcare and productivity loss costs compared with those without insomnia, but this difference did not reach statistical significance (mean difference in healthcare cost: A$142, 95% CI: −248 to 532; mean difference in productivity loss cost: A$1865, 95% CI: −3668 to 7398). For severe MDD, adults with insomnia symptoms demonstrated lower healthcare costs (mean difference: −A$746, 95% CI: −4231 to 2739) and much higher productivity loss costs (although the difference was again not statistically significant) compared with those with insomnia (mean difference: A$5845, 95% CI: −13 440 to 25 129).

**Table 5:** Subgroup analyses by depression severity levels

Depression	Insomnia	Probability of incurring costs	Costs if any incurred	Average cost per person A$ (95% CI)	Mean difference A$ (95% CI)	
		(95% CI)	A$ (95% CI)			
Healthcare cost						
Subthreshold depression	No	0.637 (0.490, 0.785)	1496 (410, 2582)	971 (387, 1555)		
	Yes	0.681 (0.612, 0.749)	2834 (2098, 3570)	1961 (1471, 2451)	990 (234, 1747)^a^	
Mild to moderate MDD	No	0.692 (0.558, 0.826)	1278 (807, 1748)	890 (563, 1217)		
	Yes	0.705 (0.660, 0.751)	1454 (1106, 1802)	1032 (775, 1289)	142 (−248, 532)	
Severe MDD	No	0.880 (0.760, 1.000)	3544 (0, 7210)	3130 (0, 6466)		
	Yes	0.755 (0.701, 0.809)	3140 (2025, 4254)	2385 (1558, 3212)	−746 (−4231, 2739)	
Probability of being employed						
Subthreshold depression	No	0.765 (0.697, 0.834)				
	Yes	0.663 (0.607, 0.720)				
Mild to moderate MDD	No	0.778 (0.684, 0.872)				
	Yes	0.745 (0.707, 0.782)				
Severe MDD	No	0.774 (0.621, 0.927)				
	Yes	0.652 (0.603, 0.701)				
Productivity loss cost for employed people						
Subthreshold depression	No	0.766 (0.662, 0.871)	18 059 (9687, 26430)	13 153 (5225, 21 081)		
	Yes	0.874 (0.834, 0.913)	21 007 (17 629, 24 384)	17 845 (14 744, 2 0945)	4692 (−3813, 13 196)	
Mild to moderate MDD	No	0.899 (0.851, 0.947)	12 214 (6945, 17 483)	10 721 (5696, 15 747)		
	Yes	0.865 (0.834, 0.897)	15 016 (11 924, 18 107)	12 586 (9676, 15 497)	1865 (−3668, 7398)	
Severe MDD	No	0.925 (0.842, 1.000)	32 977 (15 896, 50 058)	29 950 (13 263, 46 637)		
	Yes	0.948 (0.919, 0.977)	38 241 (30 433, 46 049)	35 795 (28 227, 43 362)	5845 (−13 440, 25 129)	
Societal cost						
Subthreshold depression	No	0.809 (0.707, 0.911)	12 806 (3414, 22 198)	10 463 (2504, 18 423)		
	Yes	0.869 (0.833, 0.905)	12 079 (9993, 14 166)	10 570 (8643, 12 496)	107 (−8026, 8239)	
Mild to moderate MDD	No	0.879 (0.765, 0.993)	7199 (4059, 10 340)	6361 (3609, 9113)		
	Yes	0.909 (0.887, 0.931)	8689 (6574, 10 803)	7927 (5945, 9908)	1566 (−1758, 4890)	
Severe MDD	No	0.931 (0.850, 1.000)	27 469 (0, 67 225)	25 651 (0, 63 318)		
	Yes	0.858 (0.810, 0.906)	20 083 (15 018, 25 149)	17 332 (13 016, 21 648)	−8318 (−46 242, 29 606)	

A$, Australian dollars. MDD, major depressive disorder.

a. 95% CI not including the null value (zero), indicating a statistically significant difference.

## Discussion

To the best of our knowledge, this study is the first to compare healthcare and societal costs between people with and without insomnia symptoms in patient populations with subthreshold depression and MDD using a large-scale nationally representative survey and the most reliable sources for unit costs within the Australian context. Insomnia was found to be common in both adults with subthreshold depression and those with MDD, affecting 84% of individuals, and its occurrence increased with higher severity levels of depression. Healthcare resource use was found not to be different between individuals with and without insomnia for both subthreshold depression and MDD. However, healthcare costs were significantly higher for individuals with insomnia compared with those without insomnia in the subthreshold depression population. Although the difference did not reach statistical significance, lower employment probability and reduced work productivity for employed people should be considered in adults with comorbid insomnia and depression.

Previous research has compared the healthcare resource use and healthcare costs of MDD adults who were treated with insomnia medications compared with those who did not have insomnia^25^ or did not use insomnia medications.^26^ However, it was challenging to compare our findings with those of these previous studies, as they defined the populations of interest differently. The previous studies found that adults with MDD using insomnia medications had mean healthcare costs per patient-month 2.2-fold higher than those of adults with MDD without sleep disorders (A$4377 *v*. A$1697, expressed as 2021–2022 A$, *P* < 0.001);^25^ they also had a A$7049 increase in costs (mean difference, 95% CI: A$6475 to A$7719, expressed as 2021–2022 A$) compared with adults with MDD not treated with insomnia medications.^26^ Increased costs of in-patient and emergency room visits associated with insomnia medications were raised as important issues with respect to the clinical and safety profiles of different medication classes.

We could not find other studies that estimated the economic costs of comorbid insomnia and subthreshold depression. The impact of insomnia on healthcare costs and work productivity in people with subthreshold depression should be considered in future research. As well as the significantly higher healthcare costs observed in adults with subthreshold depression with insomnia compared with those without insomnia, the average healthcare cost for individuals with comorbid subthreshold depression and insomnia was also greater than that for those with MDD with or without insomnia (A$1961 *v*. A$1686 or A$1625, respectively). Moreover, the average cost of productivity loss for employed people with comorbid subthreshold depression and insomnia was nearly equal to the cost for employed people with MDD without insomnia (A$17 845 *v*. A$16 215). Further studies are required to strengthen this evidence.

The individual effects of insomnia and depressive disorders on productivity loss have often been stated in previous studies.^2,3,8,27^ The findings of the present study supplemented this by highlighting the impact of insomnia on work productivity in people with depressive symptoms. For employed people, we observed considerable annual productivity loss costs in people with comorbid insomnia and depression (A$17 845 for subthreshold depression and A$22 045 for MDD); these were higher than those in people without insomnia symptoms (A$13 153 for subthreshold depression and A$16 215 for MDD), although the difference did not reach statistical significance.

Moreover, reduced employment in people with comorbid insomnia and depression symptoms should be considered. The proportion of those not working owing to illnesses was two-fold higher in individuals with insomnia than in those without insomnia for both subthreshold and MDD populations. As a result, the average societal cost not including productivity loss of not-employed people was nearly equal between the insomnia and non-insomnia groups for both subthreshold depression (A$10 570 *v*. A$10 463) and MDD (A$11,967 *v*. A$11 829). However, when productivity losses of people who were not working owing to their own illness were included, the mean difference between the insomnia and non-insomnia groups reached A$5478 for subthreshold depression and A$3882 for MDD. This finding was consistent with those of previous studies that also showed a significant impact of mental health conditions on employment.^28,29^

### Limitations

The generalisability of the study’s findings is restricted to the Australian population owing to differences in health system structure and financing models across different countries. Regarding the study design, the NSMHWB was a cross-sectional survey collected during 2020–2022, which limited our ability to establish prospective associations and causality. The present study focused on use of mental-health-related services and productivity loss and might thus have underestimated the economic impacts of insomnia and depression. Costs of falls or vehicle accidents were not included in this study.

Moreover, the period 2020–2022 coincided with the COVID-19 pandemic, which potentially affected the prevalence of mental conditions, healthcare accessibility and work productivity. The regression models were not adjusted for COVID-19 impact levels owing to a lack of this information in the NSMHWB data-set. Compared with prior research assessing costs associated with depression before the pandemic, the annual average healthcare cost per individual with MDD calculated in the current study (A$1625 for those without insomnia and A$1686 for those with insomnia) was higher than the mean cost (A$944, s.d. = A$2469, 2021–2022 A$) calculated by Lee et al^2^ using the NSMHWB 2007. It should be noted that the estimate in Lee et al^2^ was a weighted mean without adjustment for confounders.

The pandemic delayed the diagnosis of chronic diseases and disrupted health service use owing to social lockdowns and a shortage of healthcare workers. However, the Australian government implemented several strategies to address these issues, including expanding telehealth services under the MBS, increasing numbers of subsidised MBS services through the Better Access initiative, and allowing pharmacists to dispense up to a 1-month supply of many mental-health-related PBS medicines without a prescription if the medical need was considered urgent and the medication had been previously prescribed.^37^ Overall, the pandemic did not disrupt a pre-existing upward trend in mental-health-related expenditure, which increased from A$9.5 billion in 2013–2014 to A$10.0 billion in 2016–2017 and continued to rise during and after the pandemic to A$11.1 billion in 2019–2020, A$11.3 billion in 2020–2021, A$11.5 billion in 2021–2022 and A$12.2 billion in 2022–2023 (adjusted for inflation and referenced to 2021–2022 values).^38^ However, recent annual growth rates suggest a slight acceleration in the increase in mental-health-related expenditure post-pandemic.

In terms of defining depression severity, it should be noted that when an individual experienced multiple 12-month mental disorders, the WMH-CIDI severity level reflected the total impact of all mental disorders, not the individual impact of depressive disorder. This study did not include people without depression symptoms, because the questions to define insomnia (mentioned in the Method section) were only asked if a person experienced sadness, emptiness, depression, discouragement about life or loss of interest in most activities (subthreshold depression).

### Clinical implications

In our sample, when individuals with and without insomnia were compared, healthcare costs and service use appeared similar among adults with MDD, regardless of the severity level of depression. This suggests that depression remains the primary target for treatment. However, when depression did not meet full diagnostic criteria (i.e. subthreshold depression), healthcare costs associated with insomnia were higher in people who had both subthreshold depression and insomnia compared with those without insomnia. Although the proportion of healthcare service use between those with and without insomnia was not significantly different, the insomnia group showed a trend towards higher healthcare use. These findings align with the common conceptualisation of depression as the primary disorder and insomnia as a secondary symptom.^10,30,31^

There is evidence that insomnia is likely to impair response to depression treatment and worsen the quality of life of those experiencing depression.^32,33^ Untreated insomnia has also been shown to be associated with higher healthcare costs and MDD-related costs in adults with MDD in a US context.^34^ In addition, initial economic evaluations within a clinical trial that incorporated both costs and benefits of insomnia interventions to address comorbid depression and insomnia found that addition of insomnia interventions, including eszopiclone and cognitive–behavioural therapy for insomnia (as separate interventions), to depression treatments was very cost-effective compared with depression treatments alone.^35,36^ The findings of the present study may thus reflect the current clinical management practices for comorbid insomnia and depression. Further research is needed to understand the nature of the increased healthcare costs associated with insomnia in individuals with subthreshold depression and to ensure effective use of healthcare services in adults with comorbid insomnia and depression. However, given the strong evidence for treatment of insomnia and depression in individuals with comorbid insomnia and depression, our findings suggest a need for a change in current clinical practice to include integrated interventions for both conditions. Moreover, improvements in work productivity and employment among people with comorbid depression and insomnia symptoms should be considered. Future studies of interventions targeting these conditions should incorporate effects on work productivity and the employment-to-population ratio.

Finally, this study addressed a research gap by providing cost estimates for adults who have MDD and subthreshold depression with and without insomnia symptoms. These estimates could serve as parameter inputs for future model-based analyses of the cost-effectiveness of interventions that target insomnia and depression, thereby helping to generate more credible evidence for decision-making.

## Supporting information

Le et al. supplementary material Le et al. supplementary material

## Data Availability

This study used data extracted from the National Survey of Mental Health and Well-being (NSMHWB) 2020–2022 owned by the Australian Bureau of Statistics. Authorised users (P.H.L. and L.K.-D.L.) were granted access to the data-set on the DataLab platform. The microdata are available on request for any researchers who meet the eligibility criteria to access DataLab. More details can be found on the DataLab web page.^18^

## Supplementary material

The supplementary material is available online at https://doi.org/10.1192/bjo.2025.10077
