# Supplementary material for: Economic costs associated with insomnia in adults with subthreshold depression or major depressive disorder
Source: BJPsych Open. 2025 Jul 21;11(4):e158. doi: 10.1192/bjo.2025.10077 (PMC12303832; doi:10.1192/bjo.2025.10077)
Supplement: Le et al. supplementary material [file S205647242510077Xsup001.docx]

Economic costs associated with insomnia in adults with sub-threshold depression or major depressive disorder

**Contents**

[Figure S1. Study sample selection flowchart 2](#_Toc195568668)

[Table S1. List of analytical variables 3](#_Toc195568669)

[Table S2. Two-part model: Healthcare and societal cost 5](#_Toc195568670)

[Table S3. Logistic regression for probability of being employed and two-part hurdle model for productivity loss cost 6](#_Toc195568671)

[Table S4. Logistic regression model: Mental health-related consultation, hospitalisation, medication, absenteeism and presenteeism 7](#_Toc195568672)

[Table S5. Average annual healthcare and societal cost components associated with insomnia in people with sub-threshold depression and those with MDD derived from statistical models 8](#_Toc195568673)

[Table S6. Subgroup analysis by depression severity levels: Two-part hurdle model to calculate healthcare and societal cost 9](#_Toc195568674)

[Table S7. Subgroup analysis by depression severity levels: Logistic regression for probability of being employed and two-part hurdle model for productivity loss cost 10](#_Toc195568675)

[References 11](#_Toc195568676)

# Figure S1. Study sample selection flowchart


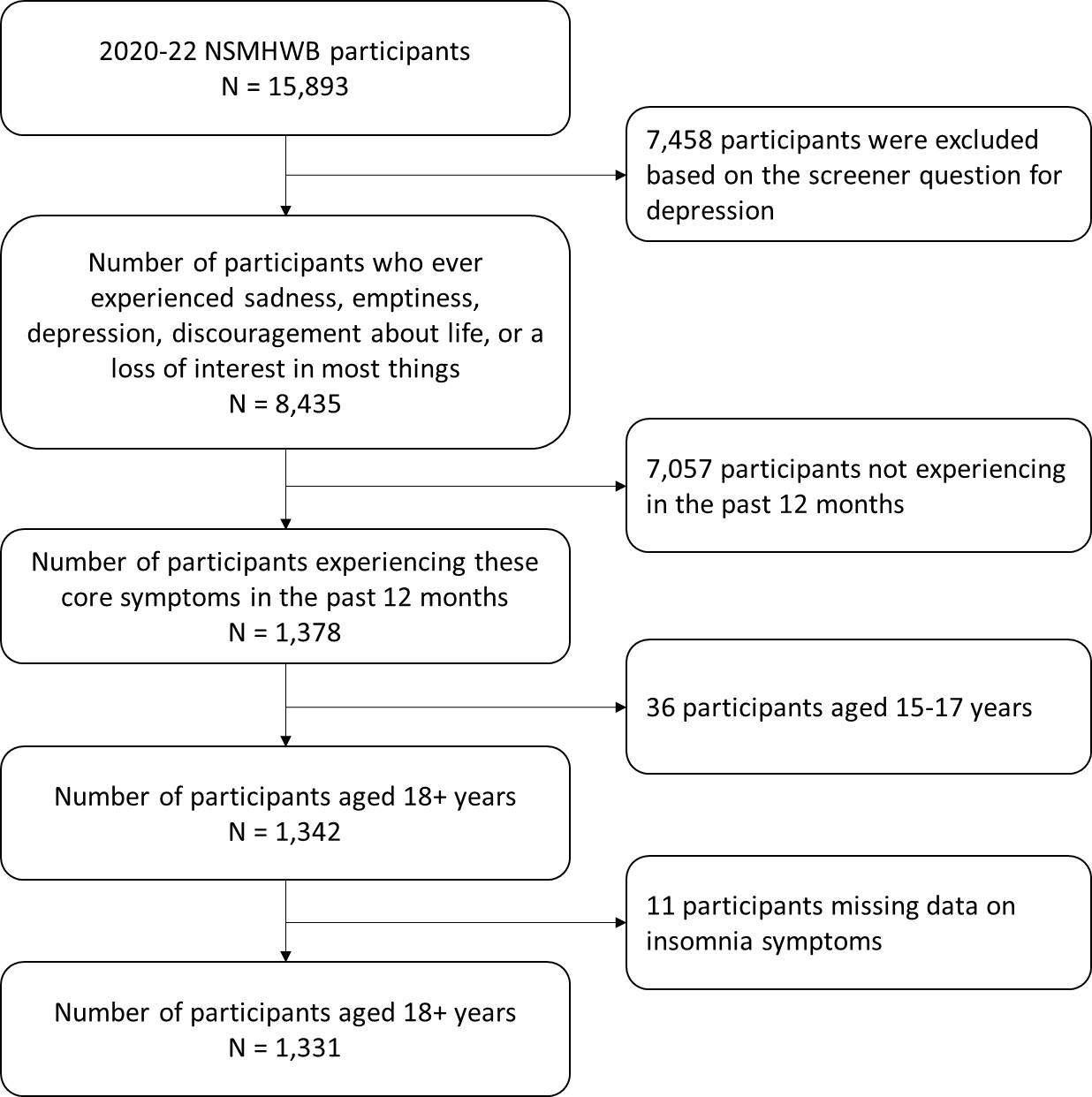


# Table S1. List of analytical variables

| **Variable** | **Description** |
| --- | --- |
| Depression | *Binary (Sub-threshold depression/ MDD)*   - Sub-threshold depression: experiencing sadness, emptiness, depression, discouragement about life, or a loss of interest in activities during the past 12 months, but not meeting the criteria for MDD; - MDD: a 12-month diagnosis for depression was based on (1) the DSM-IV criteria for a lifetime major depressive episode and major depressive disorder with the hierarchy rule applied; and (2) experiencing sufficient symptoms of these disorders in the 12 months prior to the interview. Hierarchy rules were applied, which means people were not classified as being diagnosed with a particular disorder because they met the diagnostic criteria of another mental disorder based on pre-defined rules. Details can be found elsewhere.^1^   *Subgroup analyses: Ordinal (Sub-threshold depression/Mild-Moderate MDD/Severe MDD)*: Severity levels of MDD were defined using an Australian version of WMH-CIDI. |
| Insomnia | *Binary (Yes/No)*. Self-reported difficulties in initiating or maintaining sleep. Difficulty initiating sleep was derived from a self-reported sleep latency of over 30 minutes more than half the time using a four-point question. Difficulty maintaining sleep was defined as being awake more than once a night for over 20 minutes after sleep onset (waking up in the middle of night) or waking up more than 30 minutes before the scheduled time (early morning awakening) more than half the time using two four-point questions. |
| Age | *Continuous (18 or above).*  *Binary (Adults/Older adults).* Adults were those who were aged 18-64 years, and older adults were those who were aged 65+ years. |
| Sex | *Binary (Male/Female)*. Sex at birth. |
| Indigenous status | *Binary (Yes/No)* Self-reported Aboriginal or Torres Strait Islander origin. |
| Marital status | *Binary (Partnered/Non-partnered)* Self-reported social marital status. Partnered refers to married in a registered or de facto marriage. |
| Residency status | *Binary (Australian or Permanent resident/Temporary resident)* Self-reported current residency status. |
| English as main language | *Binary (Yes/No).* Self-ported main language spoken at home was English. |
| Education level | *Binary (High/Intermediate-Low).* High level of education refers to postgraduate, graduate or bachelor's degree. |
| Employment status | *Binary (Employed/Unemployed or not in labour force).* Self-reported labour force status. |
| Weekly income in the past year | *Continuous.* Self-reported total weekly income from all sources. |
| SEIFA scores (Disadvantage, National level) | *Continuous.* Socio-Economic Indexes for Areas (SEIFA) scores: The Index of Relative Socio-Economic Disadvantage 2016 SA1, National level.  Lower scores indicate that this area has more disadvantages. |
| Comorbid physical conditions | *Binary (Yes/No).* Self-reported any chronic physical conditions including cancer, stroke, heart disease, arthritis and diabetes in the past 12 months. |
| Comorbid mental conditions | *Binary (Yes/No).* People met the criteria of a 12-month diagnosis of mental disorders other than major depressive disorder. |
| Healthcare cost | *Continuous.* A sum of costs of consultations, hospitalisation and medications for mental conditions. |
| Societal cost | *Continuous.* A sum of healthcare costs and costs due to productivity loss. |
| Productivity loss cost | *Continuous.* A sum of costs due to absenteeism and presenteeism. |
| Consultation cost | *Continuous.* Cost of mental health-related consultations delivered by general practitioners, psychiatrists, psychologists, mental health nurses, other mental health professionals, specialist doctors or surgeons, or other health professionals. Unit cost was the weighted cost of the Medicare Benefits Schedule (MBS) items adjusted by the type of health professionals and length of consultations.^2^ |
| Hospitalisation cost | *Continuous.* Cost of admissions to hospital for a mental health condition by multiplying 2021-22 National Efficient Price with National Weighted Activity Unit (A$5,597) for hospitalisation reasons.^3^ |
| Medication cost | *Continuous.* Cost of mental health-related medications*. Unit cost was weighted cost from reports by the Australian Institute of Health and Welfare (AIHW) (2023)^4^ and Pharmaceutical Benefits Scheme (PBS).^5^ |
| Absenteeism cost | *Continuous.* Cost of productivity loss from days when individuals were totally unable to work. Unit cost was the average weekly income adjusted by age and sex from the Australian Bureau of Statistics.^6^ |
| Presenteeism cost | *Continuous.* Cost of productivity loss based on interference level with the ability to work (measured using a scale of ten). Unit cost was the average weekly income adjusted by age and sex from the Australian Bureau of Statistics.^6^ |
| MDD: Major depressive disorder, WMH-CIDI: World Mental Health-Composite International Diagnostic Interview  *List of medications included: doxepin, dothiepin, fluoxetine hydrochloride, clomipramine hydrochloride, mianserin hydrochloride, moclobemide, sertraline, paroxetine, amitriptyline hydrochloride, imipramine, tranylcypromine sulphate, nortriptyline hydrochloride, phenelzine sulphate, fluvoxamine, citalopram, venlafaxine, bupropion, mirtazapine, reboxetine, escitalopram, duloxetine, desvenlafaxine, temazepam, nitrazepam, flunitrazepam, zopiclone, midazolam, olanzapine, chlorpromazine hydrochloride, risperidone, flupenthixol, prochlorperazine, haloperidol, pericyazine, lithium carbonate, paliperidone, asenapine, quetiapine, zuclopenthixol, amisulpride, aripiprazole, ziprasidone, lurasidone, diazepam, oxazepam, buspirone hydrochloride, bromazepam, dexamphetamine sulphate, methylphenidate, modafinil, atomoxetine, lisdexamfetamine | |

**Two-part hurdle models to estimate healthcare and societal costs associated with insomnia in people with major depressive disorder or sub-threshold depression**

In this study, the two-part weighted models included: Part 1 was a logistic regression model to generate probabilities of incurring the cost; and Part 2 was a Gamma-log generalised linear model (GLM) to calculate the cost if any incurred. The models were adjusted by covariates and the interaction term between insomnia and depression.

The appropriateness of gamma distribution with log link function was examined using the modified Park’s test^7^ (the slope closed to two indicates gamma distribution), the Hosmer‑Lemeshow test^8^ (p-value > 0.05), and the Pregibon link test^9^ (p-value > 0.05).

Covariates – variables considered to confound the effects of insomnia and depression on healthcare and productivity loss costs – were determined based on previous studies and included age, sex, comorbid other mental health conditions, comorbid physical conditions, education level, marital status, and household socio-economic indexes.^10-27^ To avoid model overfitting, covariate selection for each part of the model was based on bootstrapping stepwise selection (1,000 iterations) using a pre-specified cut-off of p-value <0.25.^28-31^ A covariate was chosen if its proportion of being selected was 50% or more.

Since insomnia is a diagnostic symptom of MDD and other mental disorders,^32^ multicollinearities between insomnia and depression and between insomnia and comorbid other mental health conditions were tested using the Chi-squared test and Cramer's V (Cramer's V < 0.2 indicating a small effect).^33^

# Table S2. Two-part model: Healthcare and societal cost

Part 1: logistic regression, Part 2: GLM model with Gamma distribution and natural logarithm link function

|  | **Healthcare cost** | | | | **Societal cost**  **(excluding productivity loss of not-employed people)** | | | | **Societal cost**  **(including productivity loss of not-employed people)** | | | |
| --- | --- | --- | --- | --- | --- | --- | --- | --- | --- | --- | --- | --- |
|  | **Probability of incurring costs** | | **Costs of any incurred** | | **Probability of incurring costs** | | **Costs of any incurred** | | **Probability of incurring costs** | | **Costs of any incurred** | |
| **Analytic variables** | **Coefficient**  **(95% CI)** | **p-value** | **Coefficient**  **(95% CI)** | **p-value** | **Coefficient**  **(95% CI)** | **p-value** | **Coefficient**  **(95% CI)** | **p-value** | **Coefficient**  **(95% CI)** | **p-value** | **Coefficient**  **(95% CI)** | **p-value** |
| (Intercept) | **-2.66**  **(-4.22 - -1.1)** | **0.001** | **6.98**  **(5.84 - 8.35)** | **<0.001** | -0.82  (-2.89 - 1.32) | 0.447 | **7.98**  **(6.38 - 9.67)** | **<0.001** | 0.43  (-2.02 - 3.02) | 0.740 | **9.97**  **(8.81 - 11.17)** | **0.000** |
| Insomnia: Yes | 0.2  (-0.34 - 0.74) | 0.461 | 0.64  (-0.62 - 1.65) | 0.246 | 0.46  (-0.25 - 1.13) | 0.189 | -0.06  (-0.64 - 0.47) | 0.833 | 0.4  (-0.42 - 1.16) | 0.318 | 0.36 (-0.07 - 0.75) | 0.079 |
| Depression: Yes | 0.54  (-0.12 - 1.2) | 0.113 | 0.36  (-1.01 - 1.61) | 0.569 | 0.63  (-0.25 - 1.53) | 0.161 | 0.03  (-0.63 - 0.68) | 0.916 | 0.31  (-0.65 - 1.28) | 0.528 | -0.07  (-0.55 - 0.4) | 0.775 |
| Age (centered at 18) | **0.01**  **(0.01 - 0.02)** | **0.002** | -0.01  (-0.03 - 0) | 0.121 | 0  (-0.02 - 0.01) | 0.380 | 0  (-0.01 - 0.01) | 0.409 | 0.01  (-0.01 - 0.02) | 0.257 | **0.01**  **(0.01 - 0.02)** | **<0.001** |
| Sex: Female | **0.31**  **(0.05 - 0.57)** | **0.019** | -0.45  (-0.92 - 0.01) | 0.058 | 0.28  (-0.08 - 0.64) | 0.124 | **-0.31**  **(-0.56 - -0.07)** | **0.011** | 0.08  (-0.34 - 0.5) | 0.707 | **-0.35**  **(-0.53 - -0.17)** | **<0.001** |
| Comorbid mental health condition: Yes | **0.75**  **(0.47 - 1.02)** | **<0.001** | **1.09**  **(0.54 - 1.6)** | **<0.001** | **0.38**  **(0 - 0.76)** | **0.048** | **0.33**  **(0.06 - 0.6)** | **0.013** | **0.63**  **(0.19 - 1.06)** | **0.005** | **0.37**  **(0.17 - 0.57)** | **<0.001** |
| Comorbid physical condition: Yes | **0.41**  **(0.11 - 0.71)** | **0.007** | <> | <> | <> | <> | <> | <> | <> | <> | <> | <> |
| Marital status: Partnered | <> | <> | <> | <> | **0.45**  **(0.07 - 0.84)** | **0.023** | <> | <> | 0.37  (-0.07 - 0.83) | 0.108 | <> | <> |
| Education level: Intermediate/Low | <> | <> | <> | <> | -0.28  (-0.71 - 0.14) | 0.205 | -0.16  (-0.42 - 0.09) | 0.233 | -0.31  (-0.82 - 0.18) | 0.229 | 0.09  (-0.1 - 0.29) | 0.344 |
| SEIFA (‘00) | **0.2**  **(0.06 - 0.35)** | **0.005** | <> | <> | **0.20**  **(0.01 - 0.39)** | **0.037** | **0.16**  **(0.01 - 0.3)** | **0.028** | 0.09  (-0.14 - 0.31) | 0.426 | **-0.12**  **(-0.22 - -0.02)** | **0.027** |
| Interaction term: Insomnia*Depression | -0.3  (-1.03 - 0.42) | 0.417 | -0.58  (-1.94 - 0.88) | 0.404 | -0.49  (-1.48 - 0.48) | 0.323 | 0.07  (-0.63 - 0.8) | 0.837 | -0.1  (-1.19 - 0.97) | 0.850 | -0.06  (-0.58 - 0.46) | 0.811 |
| <>: not selected to be included in the model based on covariate selection method (See Methods section) | | | | | | | | | | | | |

# Table S3. Logistic regression for probability of being employed and two-part hurdle model for productivity loss cost

|  | **Employed** | | **Cost of productivity loss (for employed people)** | | | |
| --- | --- | --- | --- | --- | --- | --- |
|  | **Probability of being employed** | | **Probability of incurring costs** | | **Costs of any incurred** | |
| **Analytic variables** | **Coefficient**  **(95% CI)** | **p-value** | **Coefficient**  **(95% CI)** | **p-value** | **Coefficient**  **(95% CI)** | **p-value** |
| (Intercept) | 1.24  (-0.63 - 3.14) | 0.195 | **1.54**  **(0.73 - 2.4)** | **<0.001** | **8.86**  **(8.34 - 9.41)** | **<0.001** |
| Insomnia: Yes | -0.55  (-1.44 - 0.29) | 0.209 | 0.79  (-0.02 - 1.58) | 0.051 | 0.15  (-0.35 - 0.62) | 0.531 |
| Depression: Yes | 0.07  (-0.76 - 0.89) | 0.871 | 1.15  (0.16 - 2.23) | 0.027 | 0.01  (-0.54 - 0.54) | 0.970 |
| Age (centered at 18) | <> | <> | **-0.03**  **(-0.05 - -0.01)** | **0.002** | **0.03**  **(0.02 - 0.04)** | **<0.001** |
| Sex: Female | -0.26  (-0.56 - 0.04) | 0.089 | -0.2  (-0.68 - 0.28) | 0.422 | -0.17  (-0.39 - 0.04) | 0.110 |
| Comorbid mental health condition: Yes | -0.07  (-0.89 - 0.73) | 0.864 | **0.64**  **(0.16 - 1.11)** | **0.009** | **0.35**  **(0.11 - 0.58)** | **0.003** |
| Comorbid physical condition: Yes | **-0.53**  **(-0.83 - -0.22)** | **0.001** | <> | <> | 0.05  (-0.19 - 0.3) | 0.688 |
| Marital status: Partnered | **0.34**  **(0.04 - 0.65)** | **0.026** | 0.38  (-0.11 - 0.88) | 0.128 | **-0.25**  **(-0.46 - -0.03)** | **0.031** |
| Education level: Intermediate/Low | **-0.69**  **(-1.01 - -0.37)** | **<0.001** | <> | <> | <> | <> |
| SEIFA (‘00) | <> | <> | <> | <> | <> | <> |
| Interaction term: Insomnia*Depression | 0.18  (-0.71 - 1.07) | 0.693 | -0.92  (-2.14 - 0.22) | 0.122 | 0.17  (-0.42 - 0.78) | 0.571 |
| <>: not selected to be included in the model based on covariate selection method (See Methods section) | | | | | | |

# Table S4. Logistic regression model: Mental health-related consultation, hospitalisation, medication, absenteeism and presenteeism

|  | **Probability of using consultations** | | **Probability of hospitalisation** | | **Probability of using medication** | | **Probability of absenteeism** | | **Probability of presenteeism** | | | |
| --- | --- | --- | --- | --- | --- | --- | --- | --- | --- | --- | --- | --- |
| **Analytic variables** | **Coefficient**  **(95% CI)** | **p-value** | **Coefficient**  **(95% CI)** | **p-value** | **Coefficient**  **(95% CI)** | **p-value** | **Coefficient**  **(95% CI)** | **p-value** | **Coefficient**  **(95% CI)** | | **p-value** |  |
| (Intercept) | **-1.92**  **(-3.41 - -0.44)** | **0.012** | -2.36  (-7.16 - 1.85) | 0.300 | **-1.92**  **(-3.42 - -0.43)** | **0.012** | -0.39  (-1 - 0.22) | 0.214 | **1.47**  **(0.73 - 2.27)** | | **<0.001** |  |
| Insomnia: Yes | 0.30  (-0.21 - 0.8) | 0.246 | 1.03  (-0.62 - 3.86) | 0.323 | 0.28  (-0.23 - 0.79) | 0.276 | **0.67**  **(0.07 - 1.27)** | **0.030** | 0.71  (-0.06 - 1.45) | | 0.066 |  |
| Depression: Yes | 0.33  (-0.26 - 0.93) | 0.271 | 0.17  (-2.4 - 3.23) | 0.896 | 0.38  (-0.22 - 0.98) | 0.216 | 0.43  (-0.26 - 1.12) | 0.220 | 0.89  (-0.02 - 1.85) | | 0.060 |  |
| Age (centered at 18) | -0.01  (-0.01 - 0) | 0.099 | -0.03  (-0.05 - 0) | 0.050 | -0.01  (-0.01 - 0) | 0.127 | **-0.02**  **(-0.03 - -0.01)** | **0.002** | **-0.02**  **(-0.03 - 0)** | | **0.015** |  |
| Sex: Female | 0.17  (-0.06 - 0.4) | 0.152 | **-0.74**  **(-1.46 - -0.03)** | **0.041** | 0.15  (-0.08 - 0.38) | 0.208 | 0.2  (-0.09 - 0.49) | 0.175 | -0.32  (-0.74 - 0.09) | | 0.130 |  |
| Comorbid mental health condition: Yes | **0.77**  **(0.53 - 1.02)** | **<0.001** | **2.00**  **(0.75 - 3.89)** | **0.009** | **0.79**  **(0.54 - 1.03)** | **<0.001** | **1.01**  **(0.71 - 1.32)** | **<0.001** | **0.46**  **(0.04 - 0.87)** | | **0.031** |  |
| Comorbid physical condition: Yes | 0.18  (-0.08 - 0.44) | 0.178 | 0.37  (-0.39 - 1.11) | 0.330 | 0.16  (-0.1 - 0.42) | 0.241 | <> | <> | <> | | <> |  |
| Marital status: Partnered | <> | <> | 0.63  (-0.1 - 1.36) | 0.090 | <> | <> | -0.07  (-0.37 - 0.24) | 0.661 | 0.26  (-0.16 - 0.69) | | 0.226 |  |
| Education level: Intermediate/Low | -0.23  (-0.48 - 0.03) | 0.088 | 0.95  (-0.03 - 2.19) | 0.087 | -0.23  (-0.49 - 0.03) | 0.085 | <> | <> | <> | | <> |  |
| SEIFA (‘00) | **0.16**  **(0.03 - 0.29)** | **0.017** | -0.33  (-0.67 - 0.02) | 0.056 | **0.16**  **(0.03 - 0.3)** | **0.016** | <> | <> | <> | | <> |  |
| Interaction term: Insomnia*Depression | -0.21  (-0.87 - 0.44) | 0.523 | -0.93  (-4.06 - 1.73) | 0.488 | -0.25  (-0.9 - 0.41) | 0.461 | -0.6  (-1.38 - 0.17) | 0.128 | -1.03  (-2.12 - 0.01) | | 0.057 |  |
| <>: not selected to be included in the model based on covariate selection method (See Methods section) | | | | | | | | | |  |  |  |

# Table S5. Average annual healthcare and societal cost components associated with insomnia in people with sub-threshold depression and those with MDD derived from statistical models

| **Depression** | **Insomnia** | **Probability of incurring costs** (Prob, 95% CI) | **Costs if any incurred**  (A$, 95% CI) | **Average cost per person**  (A$, 95% CI) | **Mean difference**  (A$, 95% CI) |  |
| --- | --- | --- | --- | --- | --- | --- |
| **Consultation and hospitalisation cost** | | | | | | |
| Sub-threshold depression | No | 0.527 (0.385, 0.669) | 1297 (647, 1947) | 725 (373, 1078) |  |  |
|  | Yes | 0.593 (0.532, 0.654) | 2896 (2207, 3585) | 1810 (1365, 2255) | 1085 (540, 1630) | * |
| MDD | No | 0.615 (0.511, 0.719) | 2324 (619, 4030) | 1503 (482, 2524) |  |  |
|  | Yes | 0.623 (0.583, 0.663) | 2350 (1756, 2944) | 1537 (1144, 1931) | 34 (-991, 1060) |  |
| **Medication cost** | | | | | | |
| Sub-threshold depression | No | 0.428 (0.318, 0.539) | 237 (166, 308) | 101 (66, 136) |  |  |
|  | Yes | 0.517 (0.449, 0.585) | 251 (224, 279) | 129 (110, 149) | 28 (-11, 67) |  |
| MDD | No | 0.520 (0.404, 0.635) | 233 (179, 287) | 120 (83, 158) |  |  |
|  | Yes | 0.511 (0.468, 0.555) | 218 (199, 236) | 111 (97, 124) | -10 (-47, 28) |  |
| **Absenteeism cost for employed people** | | | | | | |
| Sub-threshold depression | No | 0.491 (0.348, 0.634) | 13810 (7266, 20354) | 6134 (1118, 11150) |  |  |
|  | Yes | 0.640 (0.567, 0.713) | 12229 (9311, 15147) | 7289 (5461, 9118) | 1155 (-3984, 6295) |  |
| MDD | No | 0.589 (0.480, 0.697) | 9718 (5741, 13695) | 5273 (2707, 7839) |  |  |
|  | Yes | 0.603 (0.554, 0.652) | 13157 (10751, 15563) | 7333 (5896, 8770) | 2060 (-743, 4863) |  |
| **Presenteeism cost for employed people** | | | | | | |
| Sub-threshold depression | No | 0.760 (0.652, 0.868) | 9955 (4887, 15023) | 7360 (2985, 11735) |  |  |
|  | Yes | 0.863 (0.824, 0.903) | 13037 (11200, 14874) | 11071 (9374, 12768) | 3711 (-1036, 8458) |  |
| MDD | No | 0.883 (0.838, 0.928) | 12118 (7494, 16741) | 10550 (6174, 14927) |  |  |
|  | Yes | 0.846 (0.814, 0.878) | 17231 (13945, 20516) | 14316 (11325, 17307) | 3766 (-1146, 8677) |  |
| *95% CI not including the null value (zero), indicating a statistically significant difference | | | | | | |

# Table S6. Subgroup analysis by depression severity levels: Two-part hurdle model to calculate healthcare and societal cost

Part 1: logistic regression, Part 2: GLM model with Gamma distribution and natural logarithm link function

|  | **Healthcare cost** | | | | **Societal cost**  **(excluding productivity loss of not-employed people)** | | | |  |
| --- | --- | --- | --- | --- | --- | --- | --- | --- | --- |
|  | **Probability of incurring costs** | | **Costs of any incurred** | | **Probability of incurring costs** | | **Costs of any incurred** | |  |
| **Analytic variables** | **Coefficient**  **(95% CI)** | **p-value** | **Coefficient**  **(95% CI)** | **p-value** | **Coefficient**  **(95% CI)** | **p-value** | **Coefficient**  **(95% CI)** | **p-value** |  |
| (Intercept) | **-2.61**  **(-4.18 - -1.05)** | **0.001** | **7.16**  **(6.17 - 8.33)** | **<0.001** | **-0.98**  **(-3.06 - 1.16)** | **0.361** | **8.19**  **(6.66 - 9.81)** | **<0.001** |  |
| Insomnia: Yes | 0.21  (-0.33 - 0.75) | 0.440 | 0.67  (-0.39 - 1.55) | 0.157 | 0.41  (-0.3 - 1.08) | 0.241 | -0.07  (-0.63 - 0.42) | 0.775 |  |
| Depression: Mild-moderate | 0.27  (-0.43 - 0.97) | 0.455 | -0.12  (-1.36 - 1.1) | 0.841 | 0.47  (-0.45 - 1.44) | 0.325 | -0.62  (-1.29 - 0.05) | 0.061 |  |
| Depression: Severe | **1.50**  **(0.36 - 2.92)** | **0.019** | 0.90  (-0.5 - 2.51) | 0.219 | 1.1  (-0.35 - 3.16) | 0.194 | 0.72  (-0.12 - 1.66) | 0.103 |  |
| Age (centered at 18) | **0.01**  **(0 - 0.02)** | **0.003** | **-0.01**  **(-0.03 - 0)** | **0.046** | 0  (-0.01 - 0.01) | 0.740 | -0.01  (-0.01 - 0) | 0.142 |  |
| Sex: Female | **0.30**  **(0.04 - 0.56)** | **0.023** | **-0.50**  **(-0.91 - -0.1)** | **0.015** | 0.32  (-0.04 - 0.68) | 0.077 | **-0.36**  **(-0.6 - -0.14)** | **0.002** |  |
| Comorbid mental health condition: Yes | **0.70**  **(0.42 - 0.98)** | **<0.001** | **0.90**  **(0.41 - 1.36)** | **<0.001** | **0.42**  **(0.03 - 0.8)** | **0.033** | 0.16  (-0.1 - 0.42) | 0.209 |  |
| Comorbid physical condition: Yes | **0.41**  **(0.11 - 0.71)** | **0.007** | <> | <> | <> | <> | <> | <> |  |
| Marital status: Partnered | <> | <> | <> | <> | <> | <> | -0.2  (-0.43 - 0.04) | 0.093 |  |
| Education level: Intermediate/Low | <> | <> | <> | <> | -0.31  (-0.75 - 0.1) | 0.149 | -0.19  (-0.43 - 0.05) | 0.142 |  |
| SEIFA (‘00) | **0.20**  **(0.06 - 0.35)** | **0.005** | <> | <> | **0.23**  **(0.04 - 0.42)** | **0.017** | **0.17**  **(0.03 - 0.31)** | **0.012** |  |
| Interaction term:  Insomnia*Mild-moderate depression | -0.15  (-0.92 - 0.63) | 0.711 | -0.54  (-1.87 - 0.79) | 0.409 | -0.09  (-1.17 - 0.95) | 0.866 | 0.26  (-0.47 – 1.00) | 0.469 |  |
| Interaction term:  Insomnia*Severe depression | -1.11  (-2.58 - 0.09) | 0.094 | -0.80  (-2.48 - 0.7) | 0.307 | -1.23  (-3.33 - 0.3) | 0.162 | -0.24  (-1.23 - 0.66) | 0.610 |  |
| <>: not selected to be included in the model based on covariate selection method (See Methods section) | | | | | | | | | |

# Table S7. Subgroup analysis by depression severity levels: Logistic regression for probability of being employed and two-part hurdle model for productivity loss cost

|  | **Employed** | | **Cost of productivity loss (for employed people)** | | | |
| --- | --- | --- | --- | --- | --- | --- |
|  | **Probability of being employed** | | **Probability of incurring costs** | | **Costs of any incurred** | |
| **Analytic variables** | **Coefficient**  **(95% CI)** | **p-value** | **Coefficient**  **(95% CI)** | **p-value** | **Coefficient**  **(95% CI)** | **p-value** |
| (Intercept) | **-0.22**  **(-2.07 - 1.63)** | **0.813** | **1.62**  **(0.81 - 2.5)** | **<0.001** | **9.13**  **(8.62 - 9.67)** | **<0.001** |
| Insomnia: Yes | **-0.74**  **(-1.47 - -0.03)** | **0.044** | **0.83**  **(0.01 - 1.63)** | **0.044** | 0.17  (-0.33 - 0.63) | 0.478 |
| Depression: Mild-moderate | 0.09  (-0.81 - 0.99) | 0.846 | 1.09  (0.02 - 2.29) | 0.057 | -0.37  (-0.94 - 0.2) | 0.193 |
| Depression: Severe | 0.06  (-1.03 - 1.19) | 0.921 | 1.42  (-0.19 - 3.84) | 0.139 | 0.62  (-0.1 - 1.42) | 0.106 |
| Age (centered at 18) | **-0.07**  **(-0.08 - -0.06)** | **<0.001** | **-0.03**  **(-0.05 - -0.01)** | **0.002** | **0.03**  **(0.02 - 0.04)** | **<0.001** |
| Sex: Female | -0.19  (-0.5 - 0.11) | 0.224 | -0.25  (-0.74 - 0.24) | 0.323 | **-0.32**  **(-0.54 - -0.1)** | **0.003** |
| Comorbid mental health condition: Yes | -0.16  (-0.48 - 0.16) | 0.328 | **0.51**  **(0.01 - 1)** | **0.043** | 0.18  (-0.06 - 0.42) | 0.129 |
| Comorbid physical condition: Yes | **-0.57**  **(-0.88 - -0.26)** | **<0.001** | <> | <> | 0.08  (-0.17 - 0.33) | 0.522 |
| Marital status: Partnered | **0.34**  **(0.03 - 0.65)** | **0.033** | 0.41  (-0.09 - 0.92) | 0.109 | **-0.29**  **(-0.51 - -0.07)** | **0.010** |
| Education level: Intermediate/Low | **-0.69**  **(-1.01 - -0.37)** | **<0.001** | <> | <> | <> | <> |
| SEIFA (‘00) | **0.43**  **(0.26 - 0.59)** | **<0.001** | <> | <> | <> | <> |
| Interaction term: Insomnia*Mild-moderate depression | 0.47  (-0.51 - 1.46) | 0.343 | -1.17  (-2.52 - 0.07) | 0.073 | 0.04  (-0.61 - 0.69) | 0.907 |
| Interaction term: Insomnia*Severe depression | -0.13  (-1.32 - 1.02) | 0.826 | -0.43  (-2.97 - 1.45) | 0.687 | -0.02  (-0.87 - 0.76) | 0.959 |
| <>: not selected to be included in the model based on covariate selection method (See Methods section) | | | | | | |

# References

1. Australian Bureau of Statistics (ABS). *National Study of Mental Health and Wellbeing methodology*. 2024. (<https://www.abs.gov.au/methodologies/national-study-mental-health-and-wellbeing-methodology/2020-2022#health-service-utilisation> [cited Apr 2024]).

2. Australian Government Department of Health and Aged Care. *MBS Online*. 2021. (<https://www.mbsonline.gov.au/internet/mbsonline/publishing.nsf/Content/Downloads-210701> [cited Sep 2023]).

3. Independent Hospital Pricing Authority (IHPA). *Pricing Framework for Australian Public Hospital Services 2021–22*. 2021. (<https://www.ihacpa.gov.au/resources/pricing-framework-australian-public-hospital-services-2021-22> [cited Jun 2023]).

4. Australian Institute of Health and Welfare (AIHW). *Mental health-related prescriptions*. 2023. (<https://www.aihw.gov.au/mental-health/topic-areas/mental-health-prescriptions> [cited Dec 2023]).

5. The Pharmaceutical Benefits Scheme (PBS). *Report on Under Co-payment Prescriptions 2021–22 (by date of supply)*. 2022. (<https://www.pbs.gov.au/info/statistics/under-co-payment/ucp-data-report> [cited Dec 2023]).

6. Australian Bureau of Statistics (ABS). *Characteristics of Employment, Australia*. 2023. (<https://www.abs.gov.au/statistics/labour/earnings-and-working-conditions/characteristics-employment-australia/latest-release#data-item-list> [cited Dec 2023]).

7. Manning WG, Mullahy J. Estimating log models: to transform or not to transform? *J Health Econ* 2001; **20**(4): 461-94. Available from: 10.1016/s0167-6296(01)00086-8.

8. The Oxford Handbook of Economic Forecasting. Oxford University Press, 2011.

9. Pregibon D. Goodness of Link Tests for Generalized Linear Models. *Journal of the Royal Statistical Society Series C (Applied Statistics)* 1980; **29**(1): 15-4. Available from: 10.2307/2346405.

10. Hölzel L, Härter M, Reese C, Kriston L. Risk factors for chronic depression--a systematic review. *J Affect Disord* 2011; **129**(1-3): 1-13. Available from: 10.1016/j.jad.2010.03.025.

11. Dragioti E, Radua J, Solmi M, Arango C, Oliver D, Cortese S, et al. Global population attributable fraction of potentially modifiable risk factors for mental disorders: a meta-umbrella systematic review. *Molecular Psychiatry* 2022. Available from: 10.1038/s41380-022-01586-8.

12. Firth J, Solmi M, Wootton RE, Vancampfort D, Schuch FB, Hoare E, et al. A meta-review of "lifestyle psychiatry": the role of exercise, smoking, diet and sleep in the prevention and treatment of mental disorders. *World Psychiatry* 2020; **19**(3): 360-80. Available from: 10.1002/wps.20773.

13. Léger D, Morin CM, Uchiyama M, Hakimi Z, Cure S, Walsh JK. Chronic insomnia, quality-of-life, and utility scores: Comparison with good sleepers in a cross-sectional international survey. *Sleep Medicine* 2012; **13**(1): 43-51. Available from: <https://doi.org/10.1016/j.sleep.2011.03.020>.

14. Lallukka T, Sares-Jäske L, Kronholm E, Sääksjärvi K, Lundqvist A, Partonen T, et al. Sociodemographic and socioeconomic differences in sleep duration and insomnia-related symptoms in Finnish adults. *BMC Public Health* 2012; **12**(1): 565. Available from: 10.1186/1471-2458-12-565.

15. Sivertsen B, Krokstad S, Øverland S, Mykletun A. The epidemiology of insomnia: associations with physical and mental health. The HUNT-2 study. *J Psychosom Res* 2009; **67**(2): 109-16. Available from: 10.1016/j.jpsychores.2009.05.001.

16. Haario P, Rahkonen O, Laaksonen M, Lahelma E, Lallukka T. Bidirectional associations between insomnia symptoms and unhealthy behaviours. *J Sleep Res* 2013; **22**(1): 89-95. Available from: 10.1111/j.1365-2869.2012.01043.x.

17. Roy T, Lloyd CE. Epidemiology of depression and diabetes: a systematic review. *J Affect Disord* 2012; **142 Suppl**: S8-21. Available from: 10.1016/s0165-0327(12)70004-6.

18. Norman R, Church J, van den Berg B, Goodall S. Australian health-related quality of life population norms derived from the SF-6D. *Aust N Z J Public Health* 2013; **37**(1): 17-23. Available from: 10.1111/1753-6405.12005.

19. Hare DL, Toukhsati SR, Johansson P, Jaarsma T. Depression and cardiovascular disease: a clinical review. *Eur Heart J* 2014; **35**(21): 1365-72. Available from: 10.1093/eurheartj/eht462.

20. Baxter S, Sanderson K, Venn A, Otahal P, Palmer AJ. Construct validity of SF-6D health state utility values in an employed population. *Qual Life Res* 2015; **24**(4): 851-70. Available from: 10.1007/s11136-014-0823-4.

21. Metlaine A, Sauvet F, Gomez-Merino D, Elbaz M, Delafosse JY, Leger D, et al. Association between insomnia symptoms, job strain and burnout syndrome: a cross-sectional survey of 1300 financial workers. *BMJ Open* 2017; **7**(1): e012816. Available from: 10.1136/bmjopen-2016-012816.

22. Yang B, Wang Y, Cui F, Huang T, Sheng P, Shi T, et al. Association between insomnia and job stress: a meta-analysis. *Sleep Breath* 2018; **22**(4): 1221-31. Available from: 10.1007/s11325-018-1682-y.

23. Godos J, Grosso G, Castellano S, Galvano F, Caraci F, Ferri R. Association between diet and sleep quality: A systematic review. *Sleep Med Rev* 2021; **57**: 101430. Available from: 10.1016/j.smrv.2021.101430.

24. Sweetman A, Lack L, McEvoy RD, Smith S, Eckert DJ, Osman A, et al. Bi-directional relationships between co-morbid insomnia and sleep apnea (COMISA). *Sleep Med Rev* 2021; **60**: 101519. Available from: 10.1016/j.smrv.2021.101519.

25. Lerner DJ, Levine S, Malspeis S, D'Agostino RB. Job strain and health-related quality of life in a national sample. *American Journal of Public Health* 1994; **84**(10): 1580-5. Available from: 10.2105/AJPH.84.10.1580.

26. Le LK, Shih S, Richards-Jones S, Chatterton ML, Engel L, Stevenson C, et al. The cost of Medicare-funded medical and pharmaceutical services for mental disorders in children and adolescents in Australia. *PLoS One* 2021; **16**(4): e0249902. Available from: 10.1371/journal.pone.0249902.

27. Tian H, Abouzaid S, Gabriel S, Kahler KH, Kim E. Resource utilization and costs associated with insomnia treatment in patients with major depressive disorder. *The Primary Care Companion for CNS Disorders* 2012; **14**(5): 27239. Available

28. Tong S, Lu Y. Identification of Confounders in the Assessment of the Relationship between Lead Exposure and Child Development. *Annals of Epidemiology* 2001; **11**(1): 38-45. Available from: <https://doi.org/10.1016/S1047-2797(00)00176-9>.

29. Oftedal S, Glozier N, Holliday EG, Duncan MJ. Diet quality and depressive symptoms. Assessing the direction of the association in a population-based cohort study. *Journal of Affective Disorders* 2020; **274**: 347-53. Available from: <https://doi.org/10.1016/j.jad.2020.05.046>.

30. Steyerberg EW. Clinical Prediction Models: A Practical Approach to Development, Validation, and Updating. Springer-Verlag, 2009.

31. Zhou J, Williams C, Keng MJ, Wu R, Mihaylova B. Estimating Costs Associated with Disease Model States Using Generalized Linear Models: A Tutorial. *PharmacoEconomics* 2024; **42**(3): 261-73. Available from: 10.1007/s40273-023-01319-x.

32. American Psychiatric A, American Psychiatric Association DSMTF. Diagnostic and statistical manual of mental disorders : DSM-5. Arlington, VA : American Psychiatric Association, 2013.

33. Cramér H. Mathematical methods of statistics. Princeton University Press, 1946.
